# Dyslipidemia in active juvenile dermatomyositis patients

**DOI:** 10.1186/1546-0096-9-S1-P58

**Published:** 2011-09-14

**Authors:** KT Kozu, CA Silva, EF Borba, AME Sallum, VST Viana, LMA Campos

**Affiliations:** 1 Pediatric Rheumatology Unit, ChildrenÂ´s Institute, Clinical Hospital, University of São Paulo, Brazil; 2 Rheumatology Department, Clinical Hospital, University of São Paulo, Brazil

## Background

Juvenile dermatomyositis (JDM) patients may present many risk factors for dyslipidemia such as chronic inflammation and corticosteroid therapy. To our knowledge, the concomitant evaluation of lipid profile, anti-lipoprotein lipase antibody (anti-LPL), clinical and laboratory assessments, and treatment have not been performed.

## Aim

To evaluate dyslipidemia in JDM patients and healthy controls.

## Methods

25 JDM patients followed at the Pediatric Rheumatology Unit of our tertiary University Hospital were compared to 25 healthy controls according to demographic data, fasting lipoproteins, glycemia and insulin, anti-LPL antibodies and muscle enzymes. All patients were evaluated by the same pediatric rheumatologist in order to evaluate the following JDM scores: Disease Activity Score(DAS), Childhood Myositis Assessment Scale(CMAS), Manual Muscle Testing(MMT), Myositis Disease Activity Assessment Analogue Scale(MYOACT) and Myositis Intention To Treat Activity Index (MYTAX).

## Results

The mean current age of JDM was similar to controls (138.6±45.96 vs. 134±31 months, p=0.7), likewise the female gender (56% vs. 52%, p=0.5). Abnormal lipid profile was observed in 9 patients(36%) and 4(16%) controls, p=0.196. Of these JDM patients: low HDL levels were found in seven(28%), high triglycerides in four (TG)(16%), high total cholesterol in three(12%) and high LDL in one(4%). Lipodystrophy was observed in only one JDM patients with dyslipidemia. JDM patients presented higher levels of VLDL (16*vs.*13mg/dl,p=0.02), triglycerides (80*vs.*61mg/dl,p=0.011), fast insulin (8*vs.*3.9µU/ml,p=0.01), and lower glucose/insulin rate (8.8*vs.*19.7,p=0.004) compared to controls. JDM with dyslipidemia had higher AST levels(32 *vs* 24,5U/L, p=0,044), ESR(26*vs*14.5mm/1^st^ hour,p=0.006) and CRP (2.1*vs*0.4mg/dl,p=0.006) compared with patients with normal lipid profile. In addition, this group also had higher scores of DAS(6*vs.*2,p=0.008), MYOACT(0.13*vs.*0.01,p=0.012), MYTAX(0.06*vs.*0,p=0.018), and lower scores of CMAS(47*vs.*52,p=0.024) and MMT(78*vs.*80,p=0.001) compared to JDM without dyslipidemia. Positive correlations were detected between TG levels and CRP(r=0.697,p=0.001), DAS(r=0.610,p=0.001), MYOACT(r=0.661,p=0.001), MYTAX(r=0.511,p=0.008), and negative correlations between TG and CMAS(r=-0.506,p=0.009) and MMT(r=-0.535,p=0.005). Furthermore, positive correlations were found between LDL and DAS(r=0.425,p=0,034), MYOACT(r=0.534,p=0,006), MYTAX (r=0.415,p=0.039) and negative correlation with CMAS(r=-0.48,p=0,0248). However, no differences were found between JDM with and without dyslipidemia regarding body mass index, lipodystrophy, anti-LPL antibodies, and treatment (current and cumulative doses of prednisone, methotrexate and cyclosporine) (p>0.05).

## Conclusions

Dyslipidemia was observed in active JDM patients, suggesting that adequate disease control and individualized use of lipid-lowering agents may lower the risk of premature atherosclerosis in these patients.

